# Disproportionately High Rates of Burnout Among Disabled Caregivers During the COVID-19 Pandemic

**DOI:** 10.21203/rs.3.rs-4391256/v1

**Published:** 2024-05-22

**Authors:** Samantha Streuli, Imani Beckett, Marlene Flores, Vinton Omaleki, Ashkan Hassani, Tina Le, Richard Garfein, Rebecca Fielding-Miller

**Affiliations:** National Environmental Health Association; University of California San Diego; University of California San Diego; University of California San Diego; University of California San Diego; University of California San Diego; University of California San Diego; University of California San Diego

**Keywords:** burnout, caregiving, COVID-19, disability

## Abstract

**Background:**

Burnout is exhaustion caused by exposure to chronic stress. Prior to the COVID-19 pandemic, people with disabilities experienced high levels of burnout due to discrimination, barriers to accessing resources, and lack of accommodations. Caregivers have also experienced high levels of burnout during the COVID-19 pandemic.

**Background:**

While researchers have examined burnout among caregivers of disabled children, less research has focused on the experiences of disabled caregivers. We examined the association between caregiver disability and burnout during the pandemic.

**Methods:**

We distributed an online survey to caregivers of children enrolled in socially vulnerable elementary and middle schools in San Diego County, California between September and December, 2022. Our survey included demographic questions, questions about pandemic experiences, and a continuous burnout measure. We analyzed survey data to test our hypothesis that caregivers with a disability experienced higher levels of burnout than their non-disabled counterparts during the height of the COVID-19 pandemic. We used multivariable linear regression analysis adjusting for household income and caregiver education level.

**Results:**

Disabled caregivers self-reported higher levels of burnout than non-disabled caregivers (*B* = 0.72; p < 0.001) during the COVID-19 pandemic in bivariate and multivariable analyses. Caregivers with a higher household income (*B* = 0.04; p = 0.017) and more education (*B* = 0.13; p = 0.005) also reported higher levels of burnout.

**Conclusion:**

The COVID-19 pandemic exacerbated existing difficulties faced by disabled caregivers who often struggle to balance the demands of caregiving with their available resources. Targeted programs and policies are needed to support disabled caregivers during health emergencies that exacerbate existing inequities in access to resources.

## Background

The concept of burnout has been used colloquially by people in the United States (US) to describe the social problem of exhaustion caused by chronic stress since at least the 1970s [1]. While people initially used burnout to talk about labor issues, it has remained part of the broader public discourse on stress and exhaustion, and has been frequently used in recent years to describe the impact of COVID-19 on mental health [2–4]. Within the context of scholarly research, burnout has been used to understand people’s experiences with work, caregiving, disability, and more [5–7]. Burnout has been associated with factors such as lower levels of education, lower socioeconomic status, racial and ethnic marginalization, and lack of community and structural support [7, 8–11]. Although there is no current consensus among researchers on the definition, etiology, or measurement of burnout, most definitions converge on the idea that burnout results from a general imbalance between demands and resources [12]. Thus, our working definition of burnout within this article is focused on this imbalance of resources and demands.

Studies of caregiver burnout have found that caregivers experiencing burnout are more emotionally distant from their children, more physically and mentally exhausted, and experience greater feelings of inadequacy in caregiving abilities compared to those without burnout [12]. While both mothers and fathers have been found to experience caregiver burnout, there is a higher rate of caregiver burnout among mothers on average [13}. During the COVID-19 pandemic, caregivers experienced increasing demands on their time and the necessity to engage in more risk-mitigation activities. At the same time, many caregivers also lost access to social support and financial resources during the pandemic, which required them to “do more with less” and resulted in higher rates of caregiver burnout [14]. Some researchers have found that caregivers of children with disabilities report higher rates of burnout, which may be mitigated by greater social support [15, 16]. During the COVID-19 pandemic, caregivers of children with disabilities also reported increased challenges due to lack of structural and community support [11].

Disabled people in the US experience significant challenges due to factors such as systemic ableism [17]. These issues are further compounded for disabled people who are impacted by other forms of oppression due to their social identities. For example, women and people with Hispanic ancestry are more likely to be disabled in comparison to men and non-Hispanic white people [18, 19]. Disabled people are also more likely to have less education and lower household incomes, leading to particular challenges with accessing resources [20, 21]. Despite the direct impact of these stressors on disabled people, existing research predominantly focuses on disabled people as a *cause* of burnout in others (e.g. caregivers, employers) [7]. This has been a particularly prominent aspect of research on caregiver burnout, which often emphasizes the increased challenges and burnout that non-disabled caregivers experience when caring for disabled children [22, 23]. During the COVID-19 pandemic, disabled people experienced significant psychological stress due to increased exposure to illness as well as heightened institutional and interpersonal discrimination [24]. Disabled people also experienced greater challenges in accessing their basic needs during the COVID-19 pandemic [25]. Despite the increasing challenges faced by disabled people and caregivers during the pandemic, there is little current research on the impact of COVID-19 on disabled caregivers. This project seeks to understand whether disabled caregivers experienced higher rates of burnout during the COVID-19 pandemic compared to non-disabled caregivers.

## Methods

### Sample

Data included in these analyses were collected as part of the Safer at School Early Alert (SASEA) project. Between Fall 2020 and Spring 2022, SASEA researchers analyzed wastewater to track rates of COVID-19 infection in elementary and middle schools in San Diego County, California that were identified as socially vulnerable according to the California Healthy Places Index [26]. The SASEA project discontinued wastewater testing at schools in late 2022 and began to focus on providing other COVID-19 resources to socially vulnerable schools in San Diego County. Research during this period included surveying caregivers of children attending SASEA schools to elucidate their attitudes, perceptions, and experiences during the pandemic. Further project details are available elsewhere [25, 27].

The sample was drawn from 30 elementary and middle schools that had participated in the SASEA project. Every two weeks between September and December 2022, the SASEA team sent flyers to caregivers from participating SASEA schools via email with a link to a self-administered online survey. The SASEA team also printed physical copies of flyers and distributed them to schools. All caregivers were allowed to participate and those who participated were offered entry into a raffle for a $100 gift card. Surveys and flyers were available in English and Spanish. Caregivers were provided with a phone number to call if they preferred to complete the survey over the phone with a trained, bilingual (English/Spanish) research assistant. All participants provided signed or verbal consent, and all research procedures were reviewed and cleared by the [ANONYMIZED FOR REVIEW] Institutional Review Board (IRB).

### Measures

#### Primary Outcome:

Caregiver burnout was measured using a single survey item which asked, “Overall, based on your definition of burnout, how would you rate your level of burnout?” Possible answers ranged from 1–5, where 1 indicated low/no burnout (“I enjoy my daily activities. I have no symptoms of burnout.”) and 5 indicated very high burnout (“I feel completely burned out and often wonder if I can go on. I am at the point where I may need some change or may need to seek some sort of help.”). This is a validated single-item measure of burnout developed by Rohland and colleagues as an alternative to the Maslach Burnout Inventory [28]. For this analysis, burnout was treated as a continuous variable with values ranging from 1–5.

#### Primary Predictor:

Caregiver disability status was measured using the Global Activities Limitations Indicator (GALI) [29], a validated single survey item that asks participants to self-report: “For at least the past 6 months, to what extent have you or someone in your household been limited because of a health problem in the activities people usually do? Severely limited? Limited but not severely? Or not limited at all?” Participants who indicated any limitation were then asked to indicate whether the individual with limited ability was themselves, a child under age 18, an adult aged 18–65, or an adult over age 65. We created a binary variable where caregivers who identified themselves as being either “severely limited” or “limited but not severely” were categorized as “disabled” (1) and caregivers who indicated no limitations were categorized as “not disabled” (0).

#### Demographics & Potential Confounders

Caregivers were asked to report their gender as Male, Female, or Non-Binary. Because less than 1% of caregivers (n=1) reported being Non-Binary, we excluded the Non-Binary participant from analyses and dichotomized gender into Male and Female. Caregivers were asked to report their highest level of education from four possible categories: Some High School, High school, Bachelors, or Graduate Degree. Participants were asked to report their household income before taxes in 2021 on an ordinal scale ranging from Less than $15,000 to Over $100,000. Child’s race and ethnicity were based on caregiver reporting. Race categories included American Indian or Alaskan Native, Black or African American, Asian, Native Hawaiian or Pacific Islander, White, and Other. Ethnicity was coded as a binary variable for Hispanic and non-Hispanic.

### Analyses

We first conducted descriptive analysis of caregiver gender, child’s reported race and ethnicity, household income, and caregiver education level. Next, we conducted bivariate tests between our main outcome of interest (burnout), our primary predictor (caregiver disability), and hypothesized confounders.

We used Student’s t-tests to look for associations between caregiver burnout and binary variables (caregiver disability status, caregiver gender, and child’s ethnicity) and one-way Analysis of Variance (ANOVA) to look at the relationship between caregiver burnout and variables with more than two categories (household income, race, and caregiver education level) (Table 1). We then assessed associations between covariates and the primary predictor variable (caregiver disability) using chi-square analyses (Table 2).

Finally, we ran a series of ordinary least squares (OLS) regression analyses to examine relationships between burnout scores and caregiver disability adjusting for potential confounders. We performed regression validation by checking for linearity and looked at residual and fitted plots for our model to ensure that the mean of distribution errors was zero. Results of regression analyses were used to build a final multivariable model including variables significantly associated with burnout (Table 3).

## Results

### Sample Demographics

A total of 763 individuals responded to the survey, 20% of whom identified as disabled. The majority of participants (87.7%) identified as female while 12.3% identified as male. Most participants (55.8%) had a high school education or less. Seventy-eight percent of participants identified their child’s ethnicity as Hispanic. The mean burnout score for the full sample was 2.1 (SD 1.0) on a scale of 1 (low/no burnout) to 5 (high burnout).

### Bivariate Analyses

Caregivers with a disability reported significantly higher burnout scores (2.66) compared to caregivers without a disability (1.96; p < 0.001) (Table 1). Higher burnout scores were significantly associated with both higher income and higher caregiver education levels. Caregiver gender showed no significant association with burnout scores.

Caregivers without a disability were significantly more likely to report an income above the full sample mean ($35,000-$49,999) in comparison to caregivers with a disability (p = 0.02). No other covariates showed significant associations with caregiver disability status in these analyses (Table 2).

### Multivariable Regression Analysis

Using multivariable linear regression analysis to control for potential confounders, we found that caregiver disability was independently associated with higher burnout scores (*b* = 0.72; p < 0.001). Higher household income and higher levels of caregiver education also remained associated with higher rates of burnout, but other covariates did not and were excluded from the final model (Table 3).

## Discussion

We found that disabled caregivers reported higher rates of burnout during the COVID-19 pandemic than non-disabled caregivers even after controlling for potential confounders.

Previous literature on burnout in relation to disability has often focused on how being a caregiver to a disabled person can cause burnout [7, 15, 16]. However, researchers have recently begun to focus on how disabled people themselves may experience burnout, with a particular emphasis on “autistic burnout” [30]. To better understand the unique experiences of disabled people, researchers have suggested using minority stress models to examine burnout among disabled people because these models can address the imbalance between demands and resources as well as experiences of discrimination, internalized stigma, and social limitations that lead to increased burnout [31–33]. Disabled caregivers in particular may face significant demands that they are unable to meet due to lack of access to necessary support, which may increase burnout [25, 34]. While we know that the COVID-19 pandemic has increased demands upon caregivers and led to greater caregiver burnout [14], our work shows that disabled caregivers have been especially impacted during the pandemic.

Popular discourse surrounding disability often frames it as a “burden,” not only to the disabled person themself but to their broader care network, medical and social service systems, and the economy [35, 36]. This framing can lead to the perception that the disabled individual is a burden and that their basic needs are only worth meeting if they do not constitute any “undue burden” on able-bodied people [37]. Various studies on caregivers of disabled children have emphasized the additional burden of caring for a disabled child, often focusing on poor physical and mental health outcomes for caregivers [22, 23, 38]. Regardless of their intention, these approaches to disability and caregiving can contribute to stigmatizing narratives of disability as a burden, particularly when comparing caregivers of disabled children to caregivers of non-disabled children [39]. While some disabled people may indeed feel burdened by the management of their symptoms and care, disability justice approaches highlight the fact that living in an ableist society where one is faced with interpersonal microaggressions and significant structural barriers to resource navigation also has a substantial impact on the opportunities, resources, and outcomes of disabled people [35, 40]. Similarly, disability justice has emphasized that disabled people are often caregivers as well as recipients of care, which complicates the traditional narrative of disabled people as merely “burdensome” recipients of care [41]. The current research study provides further illustration of the fact that disabled caregivers have been unduly burdened during COVID-19.

It was somewhat unexpected that higher-income caregivers with more education reported more burnout in our study given that higher incomes and education can increase access to resources and reduce burnout [9]. Disabled caregivers within our sample also reported lower mean incomes than non-disabled caregivers, which we expected could contribute to burnout. One potential explanation for higher rates of burnout among higher-income caregivers during COVID-19 could be the greater number of middle to high-income caregivers working from home and taking on additional childcare during COVID-19 while continuing to work from home [42]. Additionally, higher-income caregivers may have been more likely to take on additional tasks to compensate for COVID-19 impacts on their children such as spending more time finding and coordinating alternative educational options during school closures [43]. These additional tasks may have contributed to the imbalance between resources and demands among higher-income caregivers. It is also possible that highly educated and higher income parents lacked familiarity with the already-limited assistance programs that exist to support those with fewer resources, and may have struggled with accessing them during the pandemic. More intersectional research is warranted to understand how struggles to balance resources and demands are specifically impacted by health emergencies, particularly for disabled individuals and other people who may be privileged in some areas (e.g. higher socioeconomic status) but marginalized in others.

### Limitations and Future Research

While previous research on caregiver burnout has focused on the gendered dynamics of this phenomenon [13], the sample for this study consisted mostly of women, making it difficult to produce a robust analysis of how burnout may vary according to caregiver gender. Our survey also did not include questions on relationship status or co-parenting, which could have a substantial impact on caregiver burnout [44]. Additionally, our measures of children’s race and ethnicity were based only on caregiver reporting, and we did not have a broad representation of all racial and ethnic categories. Previous research reporting racial and ethnic differences in disability and caregiver stress suggests that further analyses on a more racially and ethnically diverse sample are warranted [45, 46]. If we are to gain a better understanding of burnout and inequity, using minority stress models that also measure exposures to structural racism and other forms of discrimination may be a useful approach [7, 15, 16, 47].

There has been a substantial amount of research on measuring burnout [5–7, 44]. However, more work is needed to examine how burnout might be understood, used, and interpreted across cultural and linguistic groups and how this concept can be applied to experiences beyond work and caregiving [7, 48]. Our study consisted of only one burnout measure emphasizing the way that caregivers themselves interpreted the term “burnout.” Future research on burnout could use qualitative approaches to determine a contextually specific definition of the term prior to conducting surveys. Research could also emphasize determining a more appropriate term to examine the imbalance between resources and demands among populations of interest. Additionally, previous research on reducing caregiver burnout has focused primarily on individual caregiver behavioral changes such as emotional regulation and self-compassion to reduce burnout [49, 51]. Future studies of burnout among disabled and other marginalized caregivers should consider not only these individual behaviors, but structural ableism and racism and how this can impact caregiver burnout.

We used the Global Activities Limitations Indicator (GALI)^29^ to determine disability status. While the GALI is a useful indicator of disability status according to perceived limitations, there are other ways to measure disability that may provide more detailed information on individual experiences and specific barriers [52]. More research is needed to determine how specific disability experiences may differentially impact burnout for disabled caregivers. Future studies can also make use of minority stress models to better measure and understand the various factors that contribute to burnout among disabled caregivers [7].

## Conclusions

Disabled caregivers had higher rates of burnout than non-disabled caregivers during the COVID-19 pandemic. While disabled people are often framed as recipients of resources and care, it is important to also recognize their roles as caregivers who are impacted by interpersonal and structural ableism while trying to balance the demands of caregiving with their available resources. More programs and policies are needed to support disabled caregivers, especially during health emergencies that exacerbate existing inequities in access to resources.

## Figures and Tables

**Table 1.** Mean Burnout Scores by Covariates of Interest Among caregivers of kids enrolled in socially vulnerable schools in San Diego County

	Mean Burnout Score*	SD	p-value
**Disability Status** †			
Not Disabled	1.96	0.91	<0.001
Disabled	2.66	1.05	
**Caregiver Gender**			
Female	2.11	0.98	0.146
Male	1.96	0.97	
**Household Income** ‡			
Less than 15K:	1.87	0.91	
15–19,999K	1.86	1.09	
20–24,999K	2.16	1.26	
25–34,999K	2.20	1.03	0.001
35–49,999K	1.82	0.79	
50–74,999K	2.21	0.99	
75–99,999K	2.19	0.97	
100k & above	2.31	0.90	
**Caregiver Education Level** ‡			
Some High School	1.81	0.96	
High School	2.05	1.00	0.001
Bachelors	2.15	0.95	
Graduate	2.37	0.93	
**Child Race** ‡			
American Indian or Alaskan Native	2.33	0.58	
Black or African American	2.10	1.18	
Asian	1.95	0.81	0.028
Native Hawaiian or Pacific Islander	2.50	1.18	
White	2.19	0.98	
Other	1.94	0.96	
**Child Ethnicity** †			
Non-Hispanic	2.24	0.98	0.003
Hispanic	2.02	0.97	

* Burnout scores ranged from 1–5.

† Statistically significant difference in Student’s t-tests.

‡ Statistically significant difference in one-way ANOVA.

**Table 2.** Disability Status and Covariates of Interest Among caregivers of kids enrolled in socially vulnerable schools in San Diego County

	Not Disabled (n=612)	Disabled (n=151)	Full Sample (n=763)
**Caregiver Gender**			
Female	534 (87.3%)	135 (89.4%)	669 (87.7%)
Male	78 (12.7%)	16 (10.6%)	94 (12.3%)
**Household Income** *			
Less than 15K:	45 (7.4%)	23 (15.2%)	68 (8.9%)
15–19,999K	25 (4.1%)	10 (6.6%)	35 (4.6%)
20–24,999K	31 (5.1%)	7 (4.6%)	38 (5.0%)
25–34,999K	70 (11.5%)	20 (13.2%)	90 (11.8%)
35–49,999K	92 (15.1%)	16 (10.6%)	108 (14.2%)
50–74,999K	112 (18.4%)	20 (13.2%)	132 (17.4%)
75–99,999K	52 (8.5%)	20 (13.2%)	72 (9.5%)
100k & above	103 (16.9%)	20 (13.2%)	123 (16.2%)
**Caregiver Education Level**			
Some High School	57 (9.4%)	17 (11.3%)	74 (9.8%)
High School	281 (46.4%)	67 (44.7%)	348 (46.0%)
Bachelors	190 (31.4%)	45 (30.0%)	235 (31.1%)
Graduate	78 (12.9%)	21 (14.0%)	99 (13.1%)
**Child Race**			
American Indian or Alaskan Native	3 (0.5%)	0 (0.0%)	3 (0.4%)
Black or African American	40 (6.5%)	11 (7.3%)	51 (6.7%)
Asian	55 (9.0%	11 (7.3%)	66 (8.7%)
Native Hawaiian or Pacific Islander	8 (1.3%)	3 (2.0%)	11 (1.4%)
White	316 (51.6%)	90 (59.6%)	406 (53.2%)
Other	190 (31.0%)	36 (23.8%)	226 (29.6%)
**Child Ethnicity**			
Non-Hispanic	226 (37.8%)	62 (41.3%)	288 (38.5%)
Hispanic	372 (62.2%)	88 (58.7%)	460 (78.0%)

* Statistically significant difference in chi-square analyses at a level of p<0.05.

**Table 3.** Multivariable analysis of factors associated with burnout score among caregivers of kids enrolled in socially vulnerable schools in San Diego County

	*b*	95% CI	p-value
**Disabled Caregiver** (ref: not disabled)	0.72	0.55–0.90	<0.001
**Household Income** (ref: <15k)	0.04	0.01–0.08	0.017
**Caregiver Education Level** (ref: Some high school)	0.13	0.04–0.23	0.005

## Data Availability

To maintain participant privacy and anonymity, data are available from the corresponding author upon reasonable request.

## Abbreviations

SASEA: Safer at School Early Alert
GALI: Global Activity Limitations Indicator
ANOVA: Analysis of Variance
OLS: Ordinary Least Squares
